# Impact of ferumoxytol vs gadolinium on 4D flow cardiovascular magnetic resonance measurements in small children with congenital heart disease

**DOI:** 10.1186/s12968-022-00886-w

**Published:** 2022-11-10

**Authors:** Sarah E. Kollar, Michelle L. Udine, Jason G. Mandell, Russell R. Cross, Yue-Hin Loke, Laura J. Olivieri

**Affiliations:** 1 Division of Pediatric Cardiology, Children’s National Hospital, 111 Michigan Ave NW, WW 300, Suite 200, Washington, DC 20010 USA; 2 Division of Pediatric Cardiology, University of Rochester Medical Center, 601 Elmwood Ave, Rochester, NY 14642 USA

**Keywords:** Cardiac MRI, 4D flow, Ferumoxytol, Congenital heart disease

## Abstract

**Background:**

Cardiovascular magnetic resonance (CMR) allows for time-resolved three-dimensional phase-contrast (4D Flow) analysis of congenital heart disease (CHD). Higher spatial resolution in small infants requires thinner slices, which can degrade the signal. Particularly in infants, the choice of contrast agent (ferumoxytol vs. gadolinium) may influence 4D Flow CMR accuracy. Thus, we investigated the accuracy of 4D Flow CMR measurements compared to gold standard 2D flow phase contrast (PC) measurements in ferumoxytol vs. gadolinium-enhanced CMR of small CHD patients with shunt lesions.

**Methods:**

This was a retrospective study consisting of CMR studies from complex CHD patients less than 20 kg who had ferumoxytol or gadolinium-enhanced 4D Flow and standard two-dimensional phase contrast (2D-PC) flow collected. 4D Flow clinical software (Arterys) was used to measure flow in great vessels, systemic veins, and pulmonary veins. 4D Flow accuracy was defined as percent difference or correlation against conventional measurements (2D-PC) from the same vessels. Subgroup analysis was performed on two-ventricular vs single-ventricular CHD, arterial vs venous flow, as well as low flows (defined as < 1.5 L/min) in 1V CHD.

**Results:**

Twenty-one ferumoxytol-enhanced and 23 gadolinium-enhanced CMR studies were included, with no difference in age (2.1 ± 1.6 vs. 2.3 ± 1.9 years, p = 0.70), patient body surface area (0.50 ± 0.2 vs. 0.52 ± 0.2 m^2^, p = 0.67), or vessel diameter (11.4 ± 5.2 vs. 12.4 ± 5.6 mm, p = 0.22). Ten CMR studies with single ventricular CHD were included. Overall, ferumoxytol-enhanced 4D flow CMR measurements demonstrated less percent difference to 2D-PC when compared to gadolinium-enhanced 4D Flow CMR studies. In subgroup analyses of arterial vs. venous flows (high velocity vs. low velocity) and low flow in single ventricle CHD, ferumoxytol-enhanced 4D Flow CMR measurements had stronger correlation to 2D-PC CMR. The contrast-to-noise ratio (CNR) in ferumoxytol-enhanced studies was higher than the CNR in gadolinium-enhanced studies.

**Conclusions:**

Ferumoxytol-enhanced 4D Flow CMR has improved accuracy when compared to gadolinium 4D Flow CMR, particularly for infants with small vessels in CHD.

## Background

The management of congenital heart disease (CHD) relies on the ability to accurately assess cardiac structure, function, and hemodynamics. Cardiovascular magnetic resonance imaging (CMR) provides accurate assessments of blood flow in CHD, with multiple methods to predict prognosis and aid decision making in diseases such as coarctation of the aorta, tetralogy of Fallot, anomalous pulmonary venous return, single ventricle defects, and others [1]. For single ventricle defects, CMR can accurately assess aortopulmonary collateral burden, pulmonary artery size, and ratio of pulmonary (Qp) to systemic (Qs) blood flow [2, 3]. It is an incredibly valuable noninvasive tool.

Specific applications within CMR, such as time-resolved phase-encoded CMR imaging with velocity encoding in three directions (4D Flow), have allowed for comprehensive, accurate assessment of hemodynamics by flow quantification over an entire volume in a single acquisition [4, 5]. Traditional two-dimensional phase contrast (2D-PC) CMR allows for measurements of flow in a single 2D plane. However, a separate acquisition for each vessel of interest must be performed, incurring a significant time cost. In contrast, 4D Flow CMR data is a single acquisition which allows for post-hoc measurements of velocity and flow in any vessel or plane of interest [6]. 4D Flow CMR is routinely performed in less than 10 min and with applications including visualization of blood flow patterns, flow direction, and velocity using color-coded streamlines [7].

The precision and acquisition duration of 4D Flow CMR can be optimized by administration of exogenous blood pool contrast agents, which shorten T1 relaxivity and improve contrast-to-noise ratios (CNR) [8]. Traditional agents include gadolinium-based contrast agents. More recently, ferumoxytol has emerged as an alternative for contrast-enhanced CMR imaging. Ferumoxytol is an ultra-small superparamagnetic iron oxide nanoparticle with an intravascular half-life of 14–15 h, compared to 5–36 min for gadolinium, allowing for marked signal enhancement of the entire intravascular blood pool [9, 10]. With the extended half-life of ferumoxytol, neonates can undergo unsedated free-breathing CMR studies where repeat acquisitions can be performed if there is motion artifact without re-dosing contrast, contributing to shorter scan times compared to gadolinium [11]. Ferumoxytol is an ideal contrast agent for use with 4D Flow CMR due to the acquisition length, as it allows for increased blood pool signal for a longer period of time compared to gadolinium [12].

As noninvasive CMR is being used to assess hemodynamics of smaller babies (i.e. double outlet right ventricle, interstage single ventricles at 4–6 kg) and in smaller vessels (i.e. pulmonary vein flow to measure total pulmonary blood flow in mixing lesions), it becomes important to understand what, if any, advantages contrast agents convey in the assessment of cardiothoracic blood flow using 4D Flow CMR [13, 14]. The purpose of this study was to assess the accuracy of 4D Flow CMR (as compared with 2D-PC) in ferumoxytol-enhanced CMR studies compared to gadolinium-enhanced CMR studies in small children with CHD.

## Methods

### Study design and population

This was a single-center retrospective, Institutional Review Board-approved study of 44 sequential CMR studies performed from October 2019 to October 2021. All CMR studies were clinically indicated and protocolled to answer salient clinical questions at the time of acquisition, per lab standard, on a 1.5 T CMR scanner (Aera, Siemens Healthineers, Erlangen, Germany). All included studies had both clinically relevant 2D-PC flows performed as well as a single 4D Flow CMR retrospectively gated acquisition. Acquisition parameters are found in Table 1 for both 2D-PC and 4D Flow acquisitions for 1.3 mm isotropic (up to 7 kg) and 1.8 mm isotropic (up to 25 kg) voxels, per lab standard. Contrast agent selection was at the discretion of the overseeing imager at the time of acquisition in accordance with institutional policy, which includes reviewing an information sheet in discussion with the patient and family members regarding safety and risks of contrast. In general, very ill infants, patients who required additional non-CMR studies during one sedation, and those with any history of drug allergies did not receive ferumoxytol during this study period. Clinically appropriate velocity encoding (VENC) for 2D-PC and 4D Flow CMR varied and were chosen by the overseeing imager at the time of acquisition, and based on velocities from the most recent echocardiogram while accounting for physiologic changes anticipated with sedation, if appropriate. The overseeing imager at the time of acquisition collected 2D-PC data for all clinically relevant vessels, thus the type of vessels where flow was measured varied patient-to-patient and by diagnosis. The 4D Flow measurements were then performed for each corresponding 2D-PC acquisition. Gadolinium-enhanced studies were performed with 0.1 mmol/kg of Dotarem (Guerbet, Villepinte, France) bolus for purposes of magnetic resonance angiogram (MRA) acquisition, with 4D Flow acquisitions performed immediately following administration and 2D-PC completed within 18 ± 6 min on average. Ferumoxytol-enhanced studies were performed with 3 mg/kg of ferumoxytol (max 510 mg/dose) infused over 15 min just prior to entry to CMR suite. 2D-PC and 4D Flow CMR were completed within an average of 38 ± 8 min from the start of the scan. No studies were excluded for reasons of quality or artifacts.

**Table 1:** Lab standard for 4D flow and 2D phase contrast (2D-PC) flow acquisitions

	4D flow sequence		2D-PC flow sequence	
			Arterial	Venous
Resolution (mm)	1.3	1.8	0.6	0.6
Field of view (mm)	200	280	240	240
iPAT acceleration factor	2	2	2	2
Views per segment^a^	2–3	2–3	2–3	2–3
Matrix	72 × 160	72 × 160	192 × 192	192 × 192
Echo time (ms)	2.37–2.55	2.15–2.28	3.73	3.73
Temporal resolution (ms)	39.3–82.1	37.7–58.1	38.6	38.6
VENC (cm/s)				
Great vessels	150–400		150–350	
Branch pulmonary arteries	150–400		100–400	
Systemic veins	150–375		75–150	
Pulmonary veins	150–375		75–120	

Lab standard parameters for 4D flow performed at either 1.3 mm or 1.8 mm isotropic voxels and 2D-PC flow performed in arterial or venous structures

*VENC* velocity encoding

^a^Number of segments was 2 for RR interval < 750 ms and 3 for RR interval > 750 ms

### Flow measurements

**Fig. 1:**
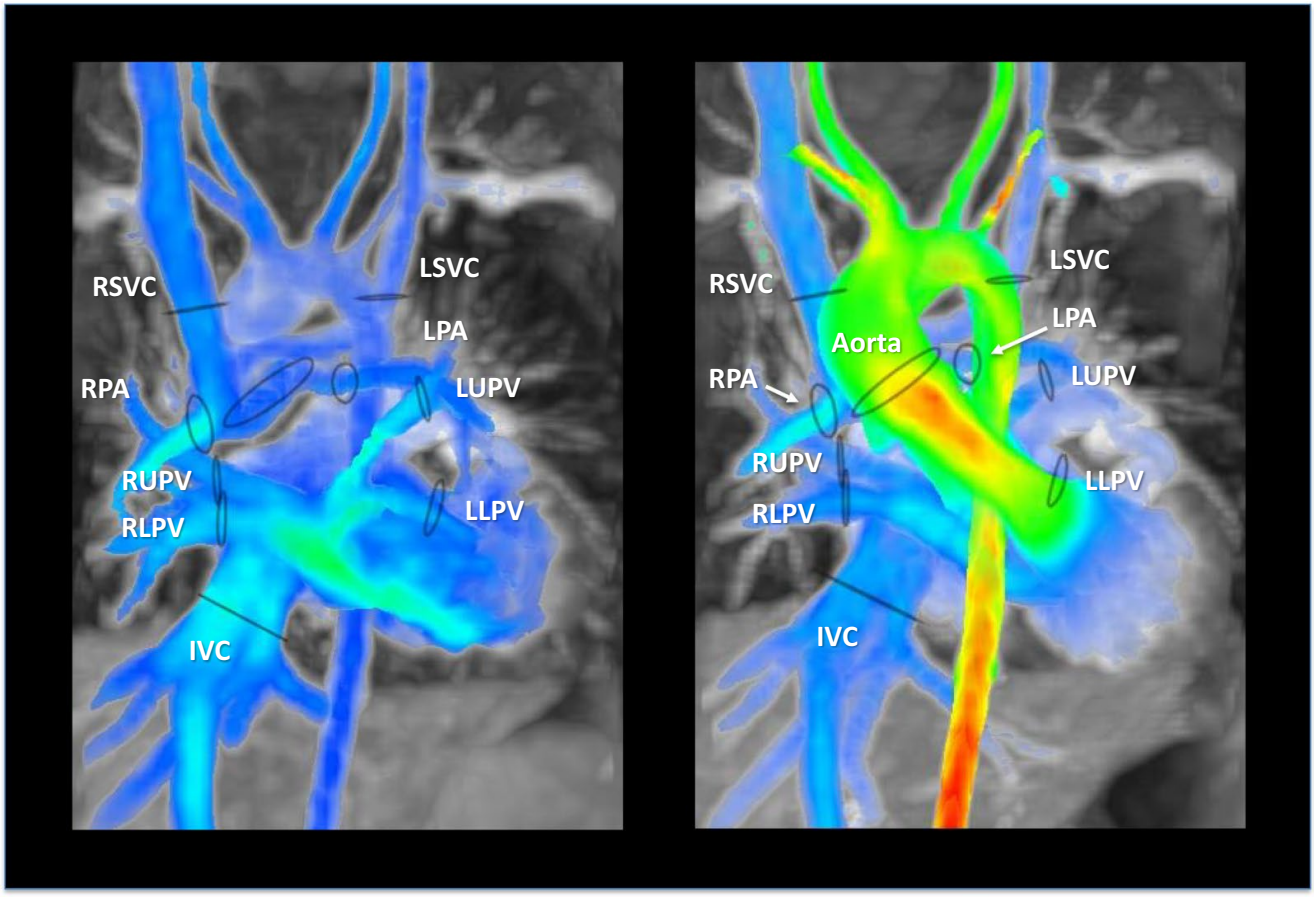
Cross-sectional view of a 5-year-old, 20 kg, single-ventricle patient status-post Glenn palliation demonstrating locations of 4D Flow quantification (black circles) positioned perpendicular to the axis of the vessel. Venous structures in diastole (left image) and arterial structures in systole (right image) were acquired in a single acquisition. Arterial and venous structures sampled include: right superior vena cava (RSVC), left superior vena cava (LSVC), left pulmonary artery (LPA), left upper pulmonary vein (LUPV), left lower pulmonary vein (LLPV), inferior vena cava (IVC), right lower pulmonary vein (RLPV), right upper pulmonary vein (RUPV), right pulmonary artery (RPA), and the aorta

Measurements of both 4D Flow and 2D-PC datasets were obtained in the great vessels (aorta, main pulmonary artery, or proximal branch pulmonary arteries), systemic veins (right or left superior vena cava and inferior vena cava), and pulmonary veins (right and left upper and lower pulmonary veins), Fig. 1. Following selection of the cohort, the patients were randomized prior to analysis, without identification of type of contrast administered for each individual study. 4D Flow measurements were performed after background-phase correction on de-identified datasets by a single investigator trained in PC measurement (Arterys Inc., San Francisco, California, USA). All 2D-PC flow measurements were performed by the non-invasive imaging cardiologist at the time of image acquisition and were reported in the medical record. A subset of 40% of studies were made available for a second, blinded reader to perform 4D Flow CMR measurements for interobserver reliability analysis. Intraobserver reliability measurements were performed at the location of the ascending aorta vs. sinotubular junction, distal right pulmonary artery vs. proximal right pulmonary artery, and high superior vena cava vs. low superior vena cava.

### Contrast-to-noise analysis

**Fig. 2:**
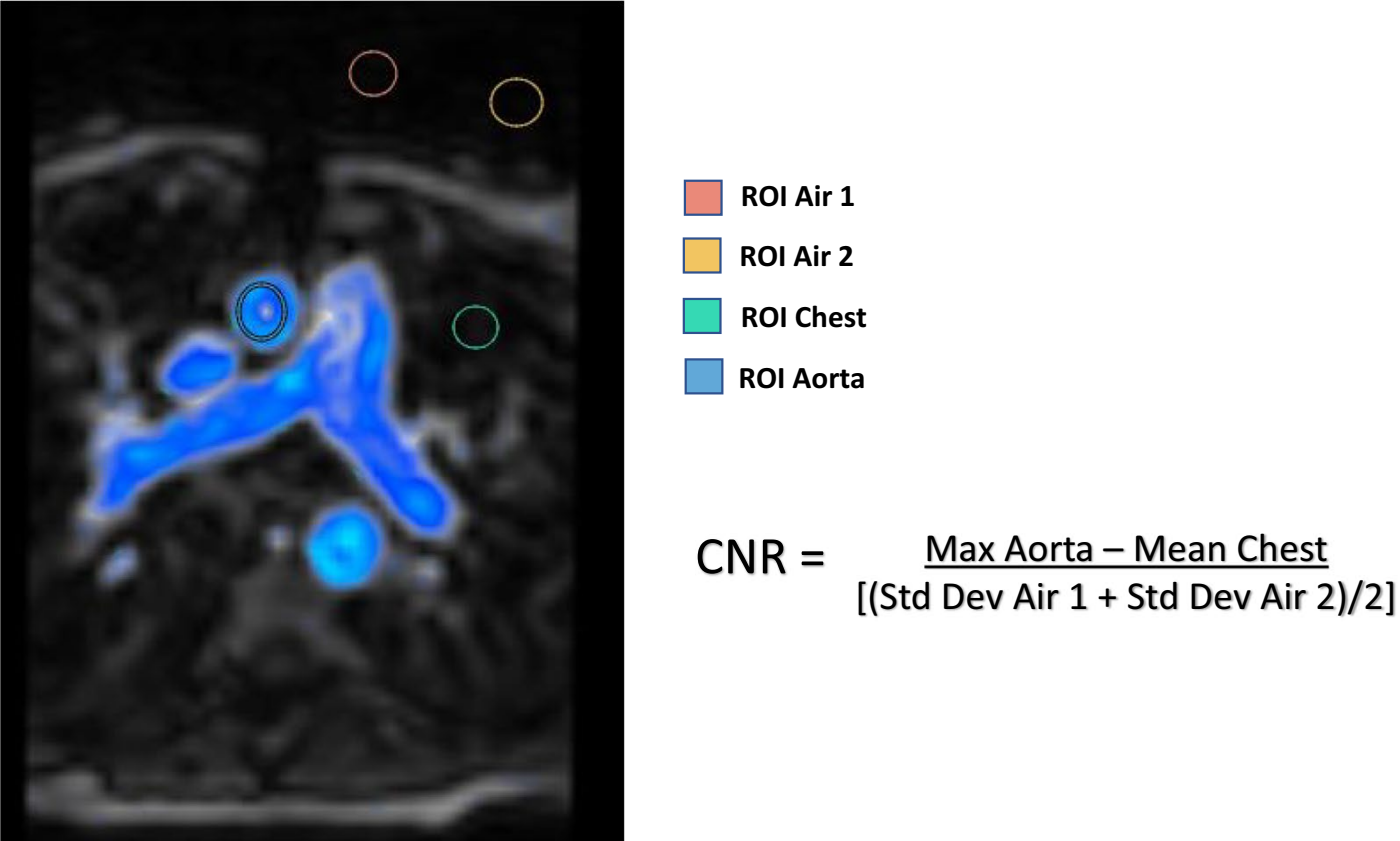
Contrast-to-noise (CNR) analysis performed by assessing multiple regions of interest (ROI), including the maximum signal in the aorta, mean signal in the chest (pleural cavity), and two separate samples to assess the standard deviation of noise in air outside the thoracic cavity

The CNR will vary with exogenous contrast administration, slice thickness and spatial resolution, which can affect accuracy of flow measurements regardless of contrast type selected. Therefore, the CNR was calculated, per 4D Flow acquisition, to assess the differences in signal intensity of the blood pool among the cohort in a similar manner to Mukai et al. [15]. Measurements were performed at end systole at the level of the aortic root in the transverse plane where corresponding 4D Flow measurements were obtained. The maximum aorta signal was obtained in the aortic root and the mean chest signal was obtained from sampling a portion of the distal lung fields away from central vasculature in the thoracic cavity. The standard deviation of noise in air was calculated from the mean of two samples outside the thoracic cavity, one anterior and midline to the chest wall and one anterior and rightward. CNR was calculated by dividing the difference between the maximum aorta signal and the mean chest signal by the standard deviation of noise in air, Fig. 2. Measurements were performed blinded to type of contrast agent administered and spatial resolution.

### Low flow and single ventricle measurements

Subgroup analysis was performed comparing two ventricle CHD patients to a subset of 10 single ventricle patients comprised of five patients with ferumoxytol and five patients with gadolinium-enhanced images. Additionally, vessels with low flows (< 1.5 L/min) were compared between the two ventricle and single ventricle cohorts. From the total cohort, the accuracy of ferumoxytol was compared to gadolinium when measuring arterial flows (aorta and pulmonary arteries) vs. venous flows (pulmonary veins and vena cavae).

### Statistical analysis

All statistical analyses were performed with Prism 8 (Graphpad, San Diego, California, USA) with the exception of interobserver analysis, which was performed with MedCalc 12.2.1.0 (MedCalce Software, Ostend, Belgium). Demographic data (including age, body surface area (BSA), and vessel diameter) and CNR for the two contrast agents were compared using an unpaired *t* test. Ferumoxytol and gadolinium-enhanced 4D Flow CMR measurements were compared as percent difference from 2D-PC flow measurements using an unpaired *t* test. Simple linear regression was used to assess 4D Flow measurements in comparison to 2D-PC flow measurements from both ferumoxytol and gadolinium-enhanced images. For all tests, a P value < 0.05 was considered statistically significant. As part of the interobserver analysis, the intraclass correlation coefficient (ICC) reflected the average level of agreement between 4D flow measurements obtained by the primary investigator and a secondary reader. Similarly, for intraobserver analysis, the ICC reflected the average level of agreement between 4D flow measurements obtained in close proximity from the same vessel by the primary investigator.

## Results

### Demographics

Measurements were obtained from 21 ferumoxytol-enhanced and 23 gadolinium-enhanced CMR studies. The patients included in this study were those with predominantly complex CHD including single ventricle anatomy, conotruncal anomalies, anomalous systemic or pulmonary venous drainage, and septal/atrioventricular valve anomalies. A subgroup analysis of 10 CMR studies focused on variability in measurements specifically in the single ventricle population.

The ferumoxytol and gadolinium administration groups were not statistically different with respect to average age (2.1 ± 1.6 vs. 2.3 ± 1.9 years), BSA (0.50 ± 0.2 m^2^ vs. 0.52 ± 0.2 m^2^), proportion of single ventricle patients (24% vs. 22%), and average vessel diameter (great vessels (16.3 ± 5 vs. 15.7 ± 5.9 mm), branch pulmonary arteries (9 ± 3.1 vs. 9.5 ± 2.7 mm), pulmonary veins (7.2 ± 1.6 vs. 7.6 ± 2 mm), and systemic veins (12.7 ± 3.9 vs. 10.7 ± 3.8 mm), Table 2. There was only one patient with significant semilunar valve disease, classified as moderate to severe truncal valve insufficiency in the gadolinium cohort. There was no statistical difference in the proportion of patients in either cohort that received moderate sedation vs. general anesthesia (p = 0.217). All 4D Flow CMR acquisitions were performed free-breathing with respiratory navigation, regardless of type of anesthesia administered. No contrast reactions were noted following administration of either ferumoxytol or gadolinium. Lastly, given the higher expected CNR, more patients given ferumoxytol were imaged with a higher resolution compared to gadolinium-based contrast.

**Table 2:** Overall demographics

	Ferumoxytol (n = 21)	Gadolinium (n = 23)	p value
Age (years)	2.1 ± 1.6	2.3 ± 1.9	0.841
Male sex	9 (43%)	13 (57%)	0.377
Height (cm)	81.8 ± 19.1	84.0 ± 18.3	0.706
Weight (kg)	11.5 ± 5.1	12.2 ± 4.8	0.599
BSA (m^2^)	0.5 ± 0.2	0.52 ± 0.2	0.667
Great vessel diameter (mm)	16.3 ± 5.0	15.7 ± 5.9	0.575
Branch pulmonary arteries (mm)	9.0 ± 3.1	9.5 ± 2.7	0.267
Pulmonary vein diameter (mm)	7.2 ± 1.6	7.6 ± 2.0	0.889
Caval diameter (mm)	12.7 ± 3.9	10.7 ± 3.8	0.115
Diagnoses			
Single ventricle patients	5 (24%)	5 (22%)	0.874
Conotruncal anomalies	7 (33%)	13 (56%)	0.129
Anomalous systemic or pulmonary venous drainage	6 (29%)	3 (13%)	0.211
Septal defects and AV valve anomalies	3 (14%)	2 (9%)	0.570
Study indication			
Eval anatomy, function, chamber size, Qp:Qs	10 (48%)	10 (43%)	0.789
Eval outflow tract obstruction/regurgitation	1 (4%)	1 (4%)	0.949
Eval branch pa stenosis/flow differential	5 (24%)	8 (35%)	0.437
Pre-Glenn/Pre-Fontan	5 (24%)	4 (18%)	0.608
Use of anesthesia			
Moderate sedation or feed and bundle	7 (33%)	12 (52%)	0.217
General anesthesia—ventilated	14 (67%)	11 (48%)	0.217
Resolution 4D (mm)			
1.30 × 1.25 × 1.25	15 (71%)	3 (13%)	< 0.001
1.80 × 1.75 × 1.75	6 (29%)	20 (87%)	< 0.001

Forty-four CMR studies from patients < 20 kg with congenital heart disease, 21 were performed with ferumoxytol and 23 with gadolinium. 10 were single ventricle patients, with 5 in the ferumoxytol cohort and 5 in the gadolinium cohort

### 4D flow vs. 2D-PC flow analysis

**Fig. 3:**
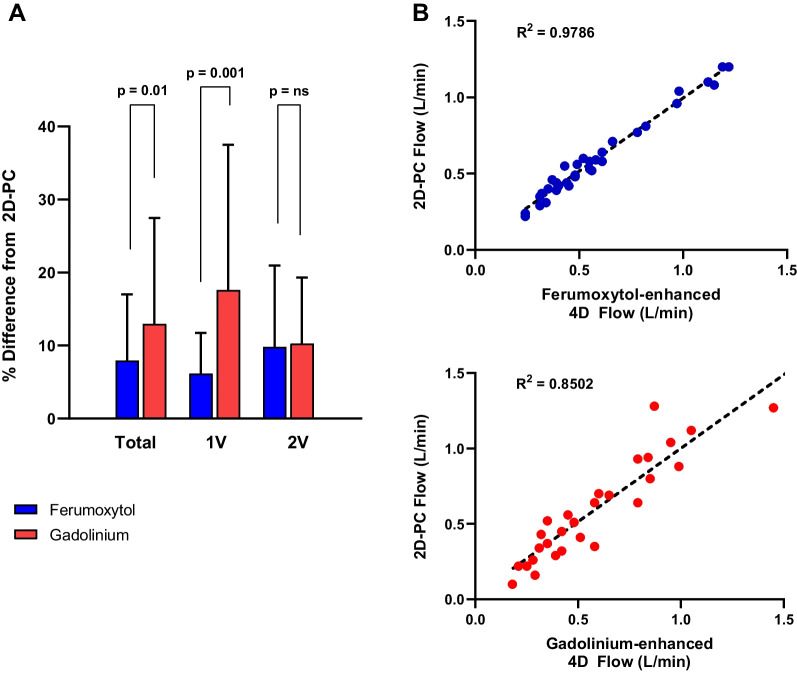
**A** Ferumoxytol-enhanced 4D flow measurements demonstrated less percent difference to 2D-PC flow measurements when compared to gadolinium-enhanced studies. **B** In subgroup analysis of low flows in 1V CHD, ferumoxytol-enhanced 4D Flow CMR measurements had stronger correlation to 2D-PC

Ferumoxytol-enhanced 4D Flow CMR measurements demonstrated less percent difference from 2D-PC flow measurements when compared to gadolinium-enhanced studies (mean difference = 7.9 vs. 13, p = 0.01). This finding was even more pronounced in the single ventricle cohort with improved accuracy of flow measurements obtained from ferumoxytol-enhanced images compared to gadolinium (mean difference = 6.1 vs. 17.6, p = 0.001), Fig. 3A. In the subgroup analysis of vessels in single ventricle patients with low flows, defined as less than 1.5 L/min, ferumoxytol-enhanced 4D Flow CMR measurements had stronger correlation to 2D-PC flow than gadolinium-enhanced measurements, Fig. 3B.

**Fig. 4:**
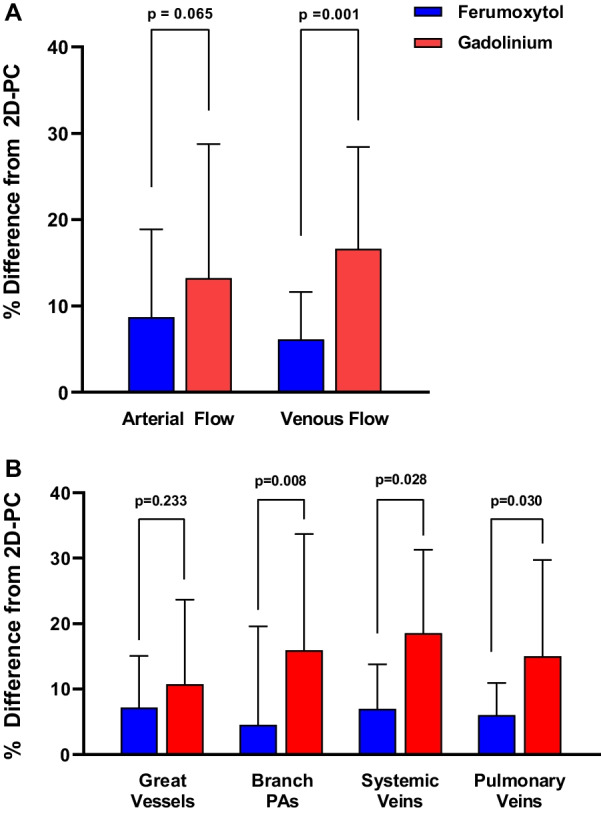
**A** In subgroup analysis of arterial (aorta and pulmonary artery) vs. venous (pulmonary veins and vena cava), ferumoxytol-enhanced 4D flow measurements had stronger correlation to 2D-PC. **B** Analysis of different vessel types demonstrated less percent difference between 4D Flow CMR and 2D-PC measurements in the ferumoxytol-enhanced studies

In the subgroup analysis of arterial (aorta and pulmonary artery) vs. venous (pulmonary veins and vena cava), ferumoxytol-enhanced 4D Flow CMR measurements demonstrated less percent difference from 2D-PC flow measurements when compared to gadolinium-enhanced studies, Fig. 4A. This finding was more pronounced in the venous flow measurements (mean difference = 6.1 vs. 16.6, p = 0.001). Additional analysis was performed to assess the difference between the various types of vessels analyzed, Fig. 4B. Ferumoxytol-enhanced 4D Flow CMR measurements demonstrated less percent difference from 2D-PC flow measurements, which was statistically significant in the branch pulmonary arteries, systemic veins, and pulmonary veins (p = 0.008, p = 0.028, and p = 0.030 respectively).

**Fig. 5:**
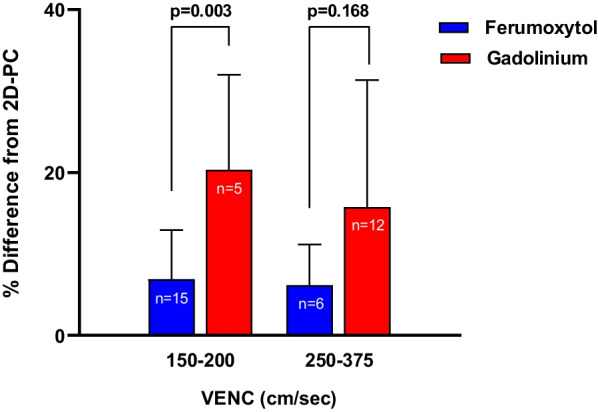
Analysis of pulmonary venous flow measurements acquired with low (150–200 cm/s) vs. high (250–375 cm/s) velocity encoding (VENC) demonstrating ferumoxytol-enhanced 4D Flow measurements had stronger correlation to 2D-PC in the low VENC cohort, but this difference was not significant in the high VENC cohort

Pulmonary venous flow measurements were isolated and analysis of 4D Flow CMR measurements acquired with low (150–200 cm/s) vs. high (250–375 cm/s) VENC was performed, Fig. 5. Ferumoxytol-enhanced 4D flow measurements demonstrated less percent difference from 2D-PC flow measurements in the low VENC cohort (mean difference = 6.8 vs. 20.3, p = 0.003), but there was no statistical difference in the high VENC cohort. In summary, all ferumoxytol-enhanced 4D flow measurements across all vessels had < 10% difference from 2D-PC measurements, compared to gadolinium which had > 10% difference.

### Contrast to noise analysis

**Fig. 6:**
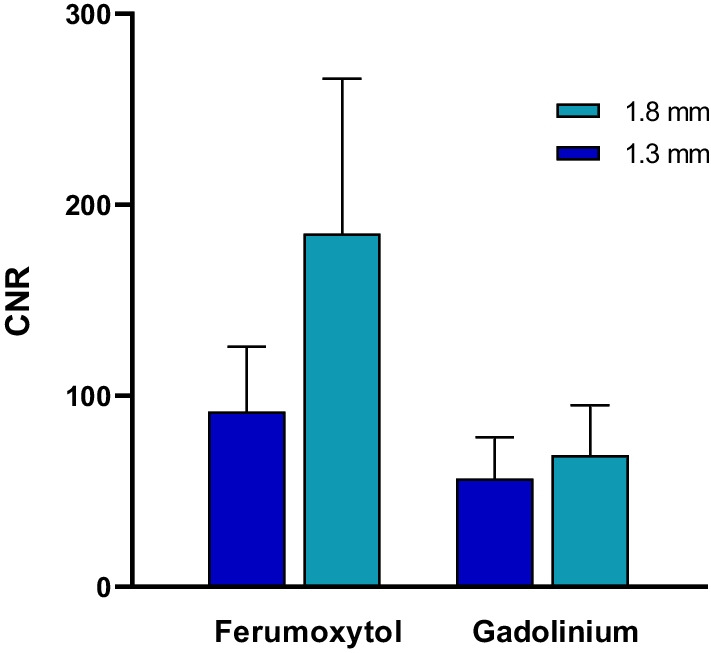
The CNR in ferumoxytol studies with a spatial resolution of 1.3 (92 ± 34, n = 14) was still higher than the CNR in gadolinium studies with a spatial resolution of 1.8 (69 ± 21, n = 20; p = 0.036)

There was a significant difference between the CNR in ferumoxytol studies with a spatial resolution of 1.3 mm (92 ± 34, n = 14) vs. 1.8 mm (185 ± 81, n = 6; p = 0.002). There was no significant difference in the CNR between gadolinium-enhanced studies performed at a spatial resolution of 1.3 mm vs. 1.8 mm. The CNR in ferumoxytol studies with a spatial resolution of 1.3 mm (92 ± 34, n = 14) was still higher than the CNR in gadolinium studies with a spatial resolution of 1.8 mm (69 ± 21, n = 20; p = 0.036), Fig. 6.

### Interobserver analysis

**Fig. 7:**
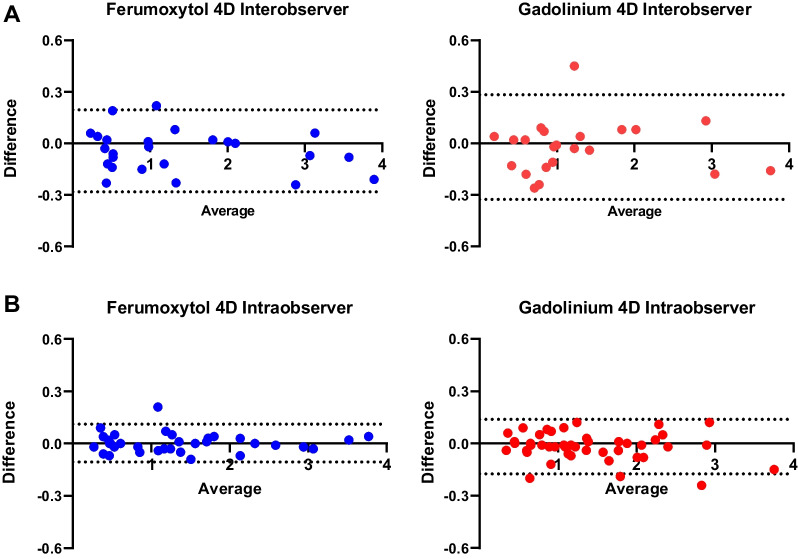
**A** Bland–Altman analysis demonstrating excellent agreement between 4D flow measurements between observers for both ferumoxytol and gadolinium-enhanced CMR. **B** Bland–Altman analysis demonstrating excellent intraobserver reproducibility of 4D Flow measurements

The ICC values reflecting the average level of agreement between 4D Flow CMR measurements obtained by the primary investigator and a secondary observer were over 95%, demonstrating excellent agreement between observers. Specifically, the ICC for ferumoxytol-enhanced 4D Flow CMR measurements was 0.997 (0.994–0.999) and similarly 0.985 (0.970–0.992) for gadolinium-enhanced 4D Flow CMR. Bland–Altman analysis in Fig. 7A demonstrates excellent agreement between observers for both contrast agents. The ICC values reflecting the average level of agreement between 4D Flow CMR measurements obtained by the primary investigator were over 95%, demonstrating excellent agreement. Specifically, the ICC for ferumoxytol-enhanced 4D Flow CMR measurements was 0.999 (0.998–0.999) and similarly 0.998 (0.995–0.999) for gadolinium-enhanced measurements. Bland–Altman analysis in Fig. 7B demonstrates excellent agreement between repeat measurements sampled in selected vessels by the primary investigator.

## Discussion

This study demonstrates excellent accuracy of 4D Flow CMR measurements (compared to 2D-PC flow measurements) in children less than 20 kg with CHD. Additionally, ferumoxytol-enhanced studies provide outstanding 4D Flow accuracy, and this is demonstrated most significantly in small children with single ventricles, small vessels, and low-velocity vessels (i.e. venous structures). These data support the use of ferumoxytol-enhanced studies in children, and particularly in children with complex CHD where it is necessary to measure flow in many thoracic vessels of varying size and peak velocities. Further, ferumoxytol-enhanced 4D Flow CMR studies had higher CNR than gadolinium-enhanced studies, even with a smaller spatial resolution.

The accuracy of measuring both intracardiac and extracardiac flows is paramount in the surgical planning of patients with complex CHD. This is especially true in neonates where measurements are performed in small-caliber vessels with low flow rates, thus requiring high spatial resolution and optimization of CNR to maintain accuracy. For example, in single ventricle patients, quantification of AP collateral burden requires flow measurements in vessels of varying size, including the branch pulmonary arteries, pulmonary veins, inferior and superior vena cava, and aortic outflow. The mean difference of ferumoxytol-enhanced 4D Flow CMR measurements in the single ventricle cohort was 6.1, in comparison to 17.6 for 2D-PC flow measurements (p = 0.001). When measuring small vessels in patients < 20 kg, these differences in flow measurements may become compounded and could become clinically significant when performing measurements of Qp:Qs, differential pulmonary blood flow, etc. Current research has even proposed utilizing hemodynamic data collected solely from CMR, instead of performing the traditional pre-Fontan cardiac catheterization [16]. 4D Flow CMR has proven to be accurate in assessment of blood flow and shunt quantification performed at multiple levels of the vascular tree, including valves, main arteries, and peripheral vessels with good intraclass agreement between observers [17].

Flow magnitudes in the CHD population are often less than 2 L/min and may require repeat acquisitions with adjustment of VENC due to aliasing of blood flow [18]. VENC adjustments are possible with 2D-PC CMR, however, standard 4D Flow CMR acquisitions require a single VENC, typically set to 1.5 m/s [19]. The average flow of all 170 vessels (arterial and venous) measured in our study was 1.3 ± 0.9 L/min and mean vessel size was 11.8 ± 5.3 mm. With vessels of this caliber, higher spatial resolution is desired, however, there are limitations of spatial resolution with gadolinium-enhanced 4D Flow CMR studies. With improved spatial resolution, there is a concordant decrease in CNR and signal-to-noise ratio (SNR), thus the clinician must balance the benefit between these two factors. This study demonstrated improved CNR with use of ferumoxytol at a spatial resolution of 1.8 mm, but also noted the CNR was still higher than what could be achieved by gadolinium at a resolution of 1.3 mm.

Ferumoxytol has many advantages over gadolinium, including its extended intravascular half-life of approximately 15 h, compared to gadolinium which is closer to 29 min [11]. The extended half-life allows for repeat scans if needed without re-dosing of the contrast agent. Gadolinium has some advantages over ferumoxytol in that there is risk of anaphylaxis associated with ferumoxytol, although this was not observed in our cohort [20]. Post-market surveillance of approximately 1.2 million doses of ferumoxytol administered from 2009 to 2015 by the United States Food & Drug Administration Adverse Event Reporting System noted 79 anaphylactic reactions with 18 fatalities, resulting in the boxed warning issued in March 2015. Of these fatalities, 24% of the patients had multiple drug allergies [21]. Additionally, there have been reports of hypotension with use of ferumoxytol, however, recent studies in pediatric patients undergoing CMR have shown the incidence of hypotension did not differ between ferumoxytol and gadolinium [22]. Gadolinium, however, carries the risk of renal toxicity, and in rare cases, nephrogenic systemic fibrosis [23]. Lastly, late gadolinium enhancement allows for assessment of myocardial tissue characteristics, which can be useful in determining both diagnosis and prognosis [24].

2D-PC CMR has proven potential as the new gold-standard for flow volume quantification in place of the formerly accepted, highly variable technique of thermodilution through invasive cardiac catheterization [25]. Presently, there has been a focus on the validation of newer and more accurate quantification of blood flow through 4D Flow CMR [26, 27]. Previous studies have demonstrated that 4D Flow CMR can be performed with sufficient resolution to accurately measure ventricular volume, mass, and function that is reproducible in comparison to that of cine balanced steady-state free precession [28]. 4D Flow data can be collected in a single acquisition, whereas 2D-PC data is collected as separate acquisitions to measure the flow in each vessel of interest, leading to increased scan times and prolonged exposure to neurotoxic effects of anesthesia [29]. Prior studies have demonstrated average 4D Flow CMR scan times of 12 min, with a range from 4 to 20 min, leading to reduction in the total CMR scan time [19]. Importantly, since a single 4D Flow data set acquires both systemic and pulmonary inflow and outflow at the same time, the 4D Flow calculations are internally validated [25]. This can be beneficial in CHD patients with hemodynamic instability where time between measurements can lead to variations in blood flow or degree of shunting, causing inaccuracies in the data obtained. With the use of ferumoxytol and 4D Flow, there is potential for less utilization of 2D-PC, leading to shorter duration of CMR scans, as well as avoidance of anesthesia for breath holds.

Previous work has focused predominantly on the reproducibility of 4D Flow CMR measurements to 2D-PC CMR using a single contrast agent for comparison. There has been some investigation of 4D Flow CMR image quality using gadolinium vs. ferumoxytol, focusing on comparison of volumetric measurements [15]. Our study is the first to validate ferumoxytol as an ideal contrast agent in small infants with CHD. It allows for highly reproducible quantification of blood flow between 4D Flow and 2D-PC CMR, compared to the more traditional use of gadolinium. This study is also unique in that it focuses on the measurements obtained from CHD patients with small caliber vessels and low flow rates. Our findings support the use of ferumoxytol as a superior contrast agent to gadolinium and provide justification for use of 4D Flow for quantification of blood flow over traditional 2D-PC flow measurements. The ability to obtain accurate, reproducible measurements will help identify optimal measures of hemodynamics that correlate with favorable measures of cardiac function and favorable clinical outcomes.

### Limitations

The study is limited in that it was performed in a single center in a retrospective manner. Because the choice to administer gadolinium vs. ferumoxytol was clinical in nature, and not systematically varied, this may have introduced bias into the cohorts receiving each contrast agent. However, there were no significant differences in any clinical variable between the two cohorts, so likely any bias was minimized. The sample size was small, although large enough to demonstrate significant reproducibility of 4D Flow CMR measurements in comparison to 2D-PC flow. Implications of the small sample size may lead to type II error and failure to reject the null hypothesis. The inequal distribution of gadolinium-enhanced studies performed at a spatial resolution of 1.3 mm (n = 3) vs. 1.8 mm (n = 20) may have contributed to the insignificant difference in the CNR between these two groups. Additionally, the 4D Flow CMR measurements were performed on a single platform with customized eddy-current phase correction to minimize vendor variability. Further investigation to determine variability between different vendor platforms and different background correction methods are needed. Additionally, the 2D-PC flow measurements were performed in a clinical setting by various trained non-invasive imaging cardiologists where the spatial resolution, velocity encoding, and scanning time were determined independently based on patient-specific clinical indications. In contrast, the 4D Flow CMR measurements were performed by an independent operator in a research setting. There were undoubtedly more patients in the gadolinium cohort with a spatial resolution of 1.8 mm, which is a limitation of the retrospective nature of this study. However, our results demonstrate the improved ability to augment CNR with ferumoxytol, even at a spatial resolution of 1.3 mm in comparison to gadolinium enhanced studies at 1.8 mm.

## Conclusion

Ferumoxytol-enhanced 4D Flow CMR is a highly reliable technique and has improved accuracy when compared to gadolinium, particularly for low flow vessels in small single ventricle CHD patients. Given the improved accuracy of ferumoxytol, its extended intravascular half-life, and marked signal enhancement, it is an ideal contrast agent for use in infants with complex CHD.

## Data Availability

The datasets used and/or analyzed during the current study are available from the corresponding author on reasonable request.

## Abbreviations

4D flow: Time-resolved three-dimensional phase-contrast
2D-PC: Two-dimensional phase contrast
BSA: Body surface area
CHD: Congenital heart disease
CMR: Cardiovascular magnetic resonance
CNR: Contrast-to-noise ratio
IVC: Inferior vena cava
LLPV: Left lower pulmonary vein
LPA: Left pulmonary artery
LSVC: Left superior vena cava
LUPV: Left upper pulmonary vein
Qp: Pulmonary blood flow
Qs: Systemic blood flow
RLPV: Right lower pulmonary vein
ROI: Region of interest
RPA: Right pulmonary artery
RSVC: Right superior vena cava
RUPV: Right upper pulmonary vein
VENC: Velocity encoding
